# Application of Ternary Nanoparticles in the Heat Transfer of an MHD Non-Newtonian Fluid Flow

**DOI:** 10.3390/mi13122149

**Published:** 2022-12-05

**Authors:** Noman Sarwar, Saad Jahangir, Muhammad Imran Asjad, Sayed M. Eldin

**Affiliations:** 1 Department of Mathematics, University of Management and Technology, Lahore 54770, Pakistan; 2 Automotive Engineering Centre, University of Engineering and Technology, Lahore 54000, Pakistan; 3 Center of Research, Faculty of Engineering, Future University in Egypt, New Cairo 11835, Egypt

**Keywords:** ternary nanoparticles, hybrid nanoparticles, mono-nanoparticles, fourier’s laws, Prabhakar fractional approach, channel flow

## Abstract

This paper introduces a novel theoretical model of ternary nanoparticles for the improvement of heat transmission. Ternary nanoparticles in a heat conductor are shown in this model. Ternary nanoparticles consist of three types of nanoparticles with different physical properties, and they are suspended in a base fluid. Analytical solutions for the temperature and velocity fields are found by using the Laplace transform approach and are modeled by using a novel fractional operator. As a result, the ternary nanoparticles are identified, and an improved heat transfer feature is observed. Further experimental research on ternary nanoparticles is being carried out in anticipation of a faster rate of heat transmission. According to the graphed data, ternary nanoparticles have greater thermal conductivity than that of hybrid nanoparticles. Moreover, the fractional approach based on the Fourier law is a more reliable and efficient way of modeling the heat transfer problem than the artificial approach. The researchers were driven to create a concept of existing nanoparticles in order to boost heat transfer, since there is a strong demand in the industry for a cooling agent with improved heat transfer capabilities.

## 1. Introduction

Channel flow is crucial in a wide variety of industrial applications, such as in chemical reactors in the research industry and heat exchangers in power plants. There are numerous associated applications wherever the continuous fluid phase shows non-Newtonian stream properties, even though many procedures of real-world significance can also be characterized as two-phase flows through Newtonian behaviors in both phases. The biochemical, biomedical, and food processing industries all provide numerous examples [1]. On the scientific scale, Dippolito et al. [2] investigated the resistance of open channel flow caused by vegetation. The effects of a vortex generator’s shape on liquids and the heat transition of hybrid nanofluids in a channel were studied by Zheng et al. [3]. Asjad et al. [4] discussed a hybrid nanoparticle analysis of fractional bioconvection in a channel flow.

Enhancing the rate of heat transmission of traditional base fluids is the main problem facing modern science and technology. To enhance heat performance and cooling systems, such as in the cooling of electronic devices, heat exchangers, and automotive cooling systems, with the greatest thermal performance, temperature reduction, precise working capability, and a long life span are required. As a result, scientists and researchers are intrigued the study of how solid particles transport heat in comparison to standard base fluids [5]. After conducting numerous studies, Choi and Eastman [6] found that adding a specific type of nano-sized particle suspension to the base fluid can speed up the rate of heat transfer. This fluid is referred to as a nanofluid. Moreover, it was found in this experiment that the base fluid’s thermal transport capabilities were enhanced by a suspension of nanoparticles, which resulted in a higher stability than that in fluids containing milli-sized and micro-sized solid particles. Eastman et al. [7] showed that a normal fluid’s thermal conductivity rises by 40 when ethylene glycol is mixed with 0.3% copper nanoparticles. Asjad et al. [8] explored advancements in generalized thermal processes and nanoparticle-based transport phenomena for vertical plates. Reddy et al. [9] discussed the performance of magnesium oxide and molybdenum disulfide nanoparticles in a micropolar thermal flux model. Izadi et al. [10] discussed a three-sided cavity and the characteristics of a porous material for the transient ordinary flow of energy transformation by a nanofluid. The extended Mittag–Leffler kernel and the impact of the MHD on a fractional Casson nanofluid with fixed boundary conditions were introduced by Saeed et al. [11].

Hybrid nanoparticles are combination of two different nanoparticles with a base fluid. Babazadeh et al. [12] studied hybrid nanofluids for free convective transportation within a porous medium in the presence of an externally supplied magnetic force. A base fluid that resulted in an exponentially extending bent surface was addressed by Nadeem et al. [13]. A study by Waini et al. [14] studied a continuous, fully established assorted convection stream on a longitudinally vertical surface embedded in a permeable medium with hybrid nanoparticles. Asadi et al. [15] addressed the topic of the effect of hybrid nanofluids on system stability. The work by Huminic et al. [16] explored different types of thermal structures intended for different boundary scenarios, and they included a summary of the effects of nanofluids and hybrid nanofluids on entropy formation. Asjad et al. [17] analyzed Prabhakar’s fractional derivative and the advancement of non-Newtonian fluids that involved hybrid nanoparticles in turbulent channels.

Recently, researchers and scientists created a new class of nanoparticles by suspending three dissimilar types of nanoparticles in a pure fluid; this new class is known as ternary hybrid nanoparticles. Researchers have modified existing nanofluids in order to improve their thermal features, leading to the introduction of tri-hybrid nanoparticles, in response to the increased demand for cooling agents combined with the high thermal capabilities at the industrial level. As a result of this reasoning, more experimental studies have been carried out to improve the thermal properties of existing ternary nanoparticles by suspending three dissimilar types of solid nanoparticles in fluids [18,19]. Sahoo and Kumar [20] invented a novel correlation for evaluating the viscosity of ternary hybrid nanoparticles. The influences of temperature and particle volume concentration on the thermo-physical characteristics and rheological behaviors of aqueous ternary hybrid CuO/MgO/TiO_2_ nanoparticles were introduced by Mousavi et al. [21]. Raju et al. [22] discussed the nonlinear motions of isotropic ternary nanoparticles in thermally radiated Darcy walls of various forms and densities with extending or expanding permeability according to elementary linear regression. The stability of tri-hybrid nanoparticles in a water–ethylene glycol mixture was studied by Ramadhan et al. [23].

Fractional derivatives are essential in the mathematical modeling of practical events. It should be mentioned that fractional calculus is a topic from differentiation science, and L’Hopital introduced it in 1695. Fractional differential equations have recently gained much attention due to their numerous applications in the domains of engineering and physics. Therefore, learning how to generalize classical fluid models to fractional models is of interest for many analysts [24,25]. Some key findings on how to solve fractional differential equations were provided by Diethelm and Ford [26]. Sene [27] investigated a fractional derivative equation and the Caputo–Liouville component derivative to study the design of a second-grade fluid. The Caputo fractional derivative was used by Mozafarifarda et al. [28] to examine fractional thermal transfer equivalence for thin metallic sheets. Reyaz et al. [29] analyzed the Caputo–Fabrizio fractional derivative to assess the effects of thermal radiation and chemical reaction on MHD Casson fluid. An application of the Caputo–Fabrizio time-fractional derivative by Haq et al. [30] revealed the impact of MHD on the channel flow of a fractionally viscous fluid through a porous medium. Thabet et al. [31] used a mathematical technique that connected the presence and constancy of ABC to study the numerical results of a unique disease, COVID-19. A Prabhakar fractional method based on the generalized Fourier law was used to study the convection flow of a Casson fluid through an oscillating surface by Sarwar et al. [32]. Shah et al. [33] discussed Prabhakar-like fractional Maxwell fluids with generalized thermal transfer in natural convection.

An important class of nanoparticles, that of mono-, hybrid, and ternary nanoparticles on a two-sided vertical plate, has not been studied while using a fractional operator. To describe the channel flow problem, the Prabhakar fractional derivative was used, as it included mono-, hybrid, and ternary nanoparticles. As a result, the primary goal was to discover analytical solutions for the energy and momentum by using the Laplace transform method. The researchers were driven to create a concept of existing nanoparticles in order to boost heat transfer, since there is a strong demand in the industry for a cooling agent with improved heat transfer capabilities. Three types of nanoparticles with different physical properties were suspended as ternary nanoparticles. In order to support the experimental results, a theoretical model for ternary nanoparticles is presented in this study. The relationship between the thermo-physical properties of ternary nanoparticles is given in Table 1. The thermo-physical properties of nanoparticles and the base fluid are given in Table 2.

## 2. Mathematical Formulation for Fourier’s Law

**Figure 1:**
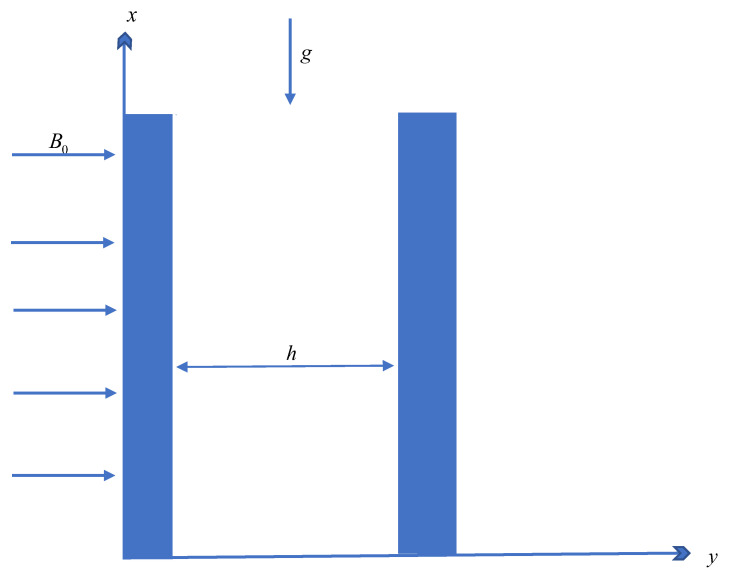
Physical model.

Let us consider an MHD convection flow through the microchannel of electrically conductive (Cu-Ag-TiO_2_) tri-hybrid nanoparticles, as shown in Figure 1, under the following constraints. The microchannel length is infinite, with width *h*. The channel is along the *x*-axis and is normal to the *y*-axis. At t=0, the temperature of the system is $T_{0}$. After $t=0^{+}$, the temperature increases from $T_{0}$ to $T_{1}$. The fluid accelerates in the *x*-direction. A magnetic field of strength $B_{0}$ works transversely to the flow direction.

The flow of electrically conductive (Cu-Ag-TiO_2_) tri-hybrid nanoparticles causes an electromotive force, which yields a current. Simultaneously, the induced magnetic field is ignored because of the hypothesis of a very small Reynolds number. Moreover, the electromagnetic force changes the intensity of the electric flux [17,18].

The momentum equation is:

(1) $$\begin{array}{c} \rho_{mnf}\left(\frac{\partial \overset{˘}{u}\left(y,t\right)}{\partial t}+\beta_{b^{*}}\overset{˘}{u}\left(y,t\right)\right)=\mu_{mnf}\frac{\partial^{2}\overset{˘}{u}\left(y,t\right)}{\partial y^{2}}-\sigma_{mnf}B_{0}^{2}\overset{˘}{u}\left(y,t\right)\\ +g{\left(\rho \beta_{T}\right)}_{mnf}\left[\overset{˘}{T}\left(y,t\right)-{\overset{˘}{T}}_{0}\right]. \end{array}$$

The energy equation is:

(2) $$\begin{array}{c} {\left(\rho C_{p}\right)}_{mnf}\frac{\partial \overset{˘}{T}\left(y,t\right)}{\partial t}=-\frac{\partial \overset{˘}{q}\left(y,t\right)}{\partial y}. \end{array}$$

The generalized Fourier law for thermal flux is as follows:

(3) $$\begin{array}{c} \overset{˘}{q}\left(y,t\right)=-k_{mnf}\;{}^{C}D_{\alpha ,\beta ,a}^{\gamma }\;\frac{\partial \overset{˘}{T}\left(y,t\right)}{\partial y}, \end{array}$$

where the definition of the regularized Prabhakar derivative is ${}^{C}D_{\alpha ,\beta ,a}^{\gamma }$ and is defined as in [27,28].

For (1)–(3), we consider the following initial and boundary conditions [37]:

(4) $$\begin{array}{c} \overset{˘}{u}\left(y,0\right)=0,\;\overset{˘}{T}\left(y,0\right)=T_{0},\;as,\;y\in \left[0,h\right], \end{array}$$

(5) $$\begin{array}{c} \overset{˘}{u}\left(0,t\right)=0,\;\overset{˘}{T}\left(0,t\right)=T_{0},\;as,\;t\geq 0, \end{array}$$

(6) $$\begin{array}{c} \overset{˘}{u}\left(h,t\right)=0,\;\overset{˘}{T}\left(h,t\right)=T_{1}. \end{array}$$

By introducing dimensionless variables, we get

(7) $$\begin{array}{c} \tau =\frac{\nu_{f}}{h^{2}}t,\;\;Y=\frac{y}{h},\;\overset{˘}{V}=\frac{h}{\nu_{f}}\overset{˘}{u},\;\;{\overset{˘}{q}}_{0}=\frac{{\overset{˘}{k}}_{mnf}\left({\overset{˘}{T}}_{1}-{\overset{˘}{T}}_{0}\right){\overset{˘}{u}}_{0}}{\nu_{mnf}},\;{\overset{˘}{q}}^{*}=\frac{\overset{˘}{q}}{{\overset{˘}{q}}_{0}},\;\;{\overset{˘}{T}}^{*}=\frac{\overset{˘}{T}-{\overset{˘}{T}}_{0}}{{\overset{˘}{T}}_{1}-{\overset{˘}{T}}_{0}}. \end{array}$$

The dimensionless fundamental equations are obtained by substituting (7) into (1)–(6) and ignoring the star documentation.

The dimensionless form of the momentum equation is as follows:

(8) $$\begin{array}{c} a_{0}^{*}\left[\frac{\partial \overset{˘}{V}\left(Y,\tau \right)}{\partial \tau }+\beta_{b}^{*}\overset{˘}{V}\left(Y,\tau \right)\right]=a_{1}^{*}\frac{\partial^{2}\overset{˘}{V}\left(Y,\tau \right)}{\partial Y^{2}}-a_{2}^{*}M\overset{˘}{V}\left(Y,\tau \right)+a_{3}^{*}\mathrm{Gr}\overset{˘}{T}\left(Y,\tau \right). \end{array}$$

The dimensionless form of the energy equation is as follows:

(9) a4*PrRe∂T˘(Y,τ)∂τ=−∂q˘(Y,τ)∂Y.

The dimensionless form of the generalized Fourier law for thermal flux [27,28] is as follows:

(10) q˘(Y,τ)=−CDα,β,aγ1Re∂T˘(Y,τ)∂Y.

The dimensionless forms of the associated conditions [37] are as follows:

(11) $$\begin{array}{c} \overset{˘}{V}\left(Y,0\right)=0,\;\overset{˘}{T}\left(Y,0\right)=0,\;as,\;Y\geq h, \end{array}$$

(12) $$\begin{array}{c} \overset{˘}{V}\left(0,\tau \right)=0,\;\;\overset{˘}{T}\left(0,\tau \right)=0,\;as,\;\tau \geq 0, \end{array}$$

(13) $$\begin{array}{c} \overset{˘}{V}\left(1,\tau \right)=0,\;\;\overset{˘}{T}\left(1,\tau \right)=1. \end{array}$$

where the variables are as follows:

Pr=(μCp)fkf,βb*=βb*h2νf,Gr=g(βT)fh3(T˘1−T˘0)νf2,M=σfh2B02μf,Re=u˘0hνmnf,a0*=1−(ϕmnf)+ϕCu(ρ)Cu+ϕAg(ρ)Ag+ϕTio2(ρ)Tio2ρf,a1*=1(1−(ϕCu+ϕAg+ϕTio2))2.5,a2*=σmnfσf,a3*=1−(ϕmnf)+ϕCu(ρβT)Cu+ϕAg(ρβT)Ag+ϕTio2(ρβT)Tio2(ρβT)f,a4*=1−(ϕmnf)+ϕCu(ρCp)Cu+ϕAg(ρCp)Ag+ϕTio2(ρCp)Tio2(ρCp)f,λmnf=kmnfkf,B0=a4*Pr,B1=a0*a1*,B2=a2*M+a0*βb*a1*,B3=a3*Gra1*,B4=B3B2.

## 3. Results of the Problem for Fourier’s Law

This section deals with the solution of the temperature and velocity fields with the Laplace transform method.

### 3.1. Outcome for the Temperature Field

By applying the Laplace transform to Equations (9) and (10) with conditions ${\left(12\right)}_{2}$ and ${\left(13\right)}_{2}$ and by utilizing the Prabhakar fractional derivative, for the temperature field, we get

(14) B0ResT˘¯(Y,s)=−∂q˘¯(Y,s)∂Y,

and

(15) q˘¯(Y,s)=−(1−as−α)γsβ1Re∂T˘¯(Y,s)∂Y.

By introducing Equation (15) into Equation (14), we get the following homogeneous differential equation:

(16) $$\begin{array}{c} \frac{\partial^{2}\bar{\overset{˘}{T}}\left(Y,s\right)}{\partial Y^{2}}-\frac{B_{0}s\bar{\overset{˘}{T}}\left(Y,s\right)}{{\left(1-as^{-\alpha }\right)}^{\gamma }s^{\beta }}=0, \end{array}$$

which satisfies the following limitations:

(17) $$\begin{array}{c} \bar{\tilde{T}}\left(0,s\right)=0,\;\;\;\;\;\bar{\tilde{T}}\left(1,s\right)=\frac{1}{s}. \end{array}$$

The general solution of Equation (16) with Equation (17) is as follows:

(18) $$\begin{array}{c} \bar{\overset{˘}{T}}\left(Y,s\right)=\frac{1}{s}\left[\frac{sinhY\sqrt{\frac{B_{0}s}{{\left(1-as^{-\alpha }\right)}^{\gamma }s^{\beta }}}}{sinh\sqrt{\frac{B_{0}s}{{\left(1-as^{-\alpha }\right)}^{\gamma }s^{\beta }}}}\right]. \end{array}$$

It is important that Equation (18) can be written in the equivalent form:

(19) $$\begin{array}{c} \bar{\overset{˘}{T}}\left(Y,s\right)=\frac{1}{s}\left[\sum_{m=0}^{\infty }e^{-\left(2m+1-Y\right)\sqrt{\frac{B_{o}s}{{\left(1-as^{-\alpha }\right)}^{\gamma }s^{\beta }}}}-\sum_{m=0}^{\infty }e^{-\left(2m+1+Y\right)\sqrt{\frac{B_{o}s}{{\left(1-as^{-\alpha }\right)}^{\gamma }s^{\beta }}}}\right]. \end{array}$$

The inverse Laplace transform cannot be found simply from the Laplace transform equation. As a result, Equation (19) is represented in the series as follows:

(20) $$\begin{array}{c} \bar{\overset{˘}{T}}\left(Y,s\right)=\frac{1}{s}+\sum_{m=0}^{\infty }\sum_{n=1}^{\infty }\sum_{k=0}^{\infty }\frac{{\left(Y-2m-1\right)}^{n}{\left(B_{o}\right)}^{\frac{n}{2}}{\left(a\right)}^{k}}{n!k!\;s^{\alpha k+\frac{\beta n}{2}-\frac{n}{2}+1}}\frac{\Gamma (\frac{\gamma n}{2}+k)}{\Gamma (\frac{\gamma n}{2})}+\\ \sum_{m=0}^{\infty }\sum_{p=0}^{\infty }\sum_{l=0}^{\infty }\frac{{\left(-Y-2m-1\right)}^{p}{\left(B_{o}\right)}^{\frac{p}{2}}{\left(a\right)}^{l}}{p!l!\;s^{\alpha l+\frac{\beta p}{2}-\frac{p}{2}+1}}\frac{\Gamma (\frac{\gamma p}{2}+l)}{\Gamma (\frac{\gamma p}{2})}. \end{array}$$

Using the inverse Laplace transform of Equation (20), we have

(21) $$\begin{array}{c} \overset{˘}{T}\left(Y,\tau \right)=1+\sum_{m=0}^{\infty }\sum_{n=1}^{\infty }\sum_{k=0}^{\infty }\frac{{\left(Y-2m-1\right)}^{n}{\left(B_{0}\right)}^{\frac{n}{2}}{\left(a\right)}^{k}}{n!k!\;}\frac{t^{\left(\alpha k+\frac{\beta n}{2}-\frac{n}{2}\right)}}{\Gamma (\alpha k+\frac{\beta n}{2}-\frac{n}{2}+1)}\frac{\Gamma (\frac{\gamma n}{2}+k)}{\Gamma (\frac{\gamma n}{2})}+\\ \sum_{m=0}^{\infty }\sum_{p=0}^{\infty }\sum_{l=0}^{\infty }\frac{{\left(-Y-2m-1\right)}^{p}{\left(B_{0}\right)}^{\frac{p}{2}}{\left(a\right)}^{l}}{p!l!\;}\frac{t^{\alpha l+\frac{\beta p}{2}-\frac{p}{2}}}{\Gamma (\alpha l+\frac{\beta p}{2}-\frac{p}{2}+1)}\frac{\Gamma (\frac{\gamma p}{2}+l)}{\Gamma (\frac{\gamma p}{2})}. \end{array}$$

### 3.2. Outcome for the Velocity Field

By taking the Laplace transform of Equation (8) with constraints ${\left(12\right)}_{1}$ and ${\left(13\right)}_{1}$, we attain

(22) $$\begin{array}{c} \left[\frac{\partial^{2}}{\partial Y^{2}}-B_{1}s-B_{2}\right]\bar{\overset{˘}{V}}\left(Y,s\right)=-B_{3}\bar{\overset{˘}{T}}\left(Y,s\right), \end{array}$$

which satisfies the following constraints:

(23) $$\begin{array}{c} \bar{\overset{˘}{V}}\left(0,s\right)=0,\;\;\;\;\;\bar{\overset{˘}{V}}\left(1,s\right)=0. \end{array}$$

From Equations (19), (22), and (23) we acquire the following results:

(24) $$\begin{array}{c} \bar{\overset{˘}{V}}\left(Y,s\right)=-\frac{B_{4}}{s}\left[\frac{\sum_{m=0}^{\infty }e^{-\left(2m\right)\sqrt{\frac{B_{o}s}{s^{\beta }{\left(1-as^{-\alpha }\right)}^{\gamma }}}}-\sum_{m=0}^{\infty }e^{-\left(2m+2\right)\sqrt{\frac{B_{o}s}{s^{\beta }{\left(1-as^{-\alpha }\right)}^{\gamma }}}}}{\left[1+\left(\frac{B_{1}s}{B_{2}}-\frac{B_{0}s}{B_{2}s^{\beta }{\left(1-as^{-\alpha }\right)}^{\gamma }}\right)\right]}\right]\\ \times \left[\sum_{n=0}^{\infty }e^{-\left(2n+1-Y\right)\sqrt{B_{2}+B_{1}s}}-\sum_{n=0}^{\infty }e^{-\left(2n+1+Y\right)\sqrt{B_{2}+B_{1}s}}\right]\\ +\frac{B_{4}}{s}\left[\frac{\sum_{m=0}^{\infty }e^{-\left(2m+1-Y\right)\sqrt{\frac{B_{o}s}{{\left(1-as^{-\alpha }\right)}^{\gamma }s^{\beta }}}}-\sum_{m=0}^{\infty }e^{-\left(2m+1+Y\right)\sqrt{\frac{B_{o}s}{{\left(1-as^{-\alpha }\right)}^{\gamma }s^{\beta }}}}}{\left[1+\left(\frac{B_{1}s}{B_{2}}-\frac{B_{o}s}{B_{2}{\left(1-as^{-\alpha }\right)}^{\gamma }s^{\beta }}\right)\right]}\right]. \end{array}$$

The inverse Laplace of Equation (24) can be obtained numerically by using Tzou’s and Stehfest’s algorithms [38,39].

## 4. Mathematical Formulation of the Ternary Nanoparticles for Artificial Replacement

The momentum equation is:

(25) $$\begin{array}{c} \rho_{mnf}\left(\frac{\partial \overset{˘}{u}\left(y,t\right)}{\partial t}+\beta_{b^{*}}\overset{˘}{u}\left(y,t\right)\right)=\mu_{mnf}\frac{\partial^{2}\overset{˘}{u}\left(y,t\right)}{\partial y^{2}}-\sigma_{mnf}B_{0}^{2}\overset{˘}{u}\left(y,t\right)\\ +{\left(g\beta_{T}\right)}_{mnf}\left[\overset{˘}{T}\left(y,t\right)-{\overset{˘}{T}}_{0}\right]. \end{array}$$

The generalized Fourier law for thermal flux is as follows:

(26) $$\begin{array}{c} {\left(\rho C_{p}\right)}_{mnf}\frac{\partial \overset{˘}{T}\left(y,t\right)}{\partial t}=k_{mnf}\;{}^{C}D_{\alpha ,\beta ,a}^{\gamma }\frac{\partial^{2}\overset{˘}{T}\left(y,t\right)}{\partial y^{2}}. \end{array}$$

where the definition of the regularized Prabhakar derivative is ${}^{C}D_{\alpha ,\beta ,a}^{\gamma }$ and is defined as in [27,28].

We get the results for the temperature and velocity fields by using Equations (12) and (13).

The dimensionless form of the momentum equation is:

(27) $$\begin{array}{c} a_{0}^{*}\left[{}^{C}D_{\alpha ,\beta ,a}^{\gamma }\frac{\partial \overset{˘}{V}\left(Y,\tau \right)}{\partial \tau }+\beta_{b}^{*}\overset{˘}{V}\left(Y,\tau \right)\right]=a_{1}^{*}\frac{\partial^{2}\overset{˘}{V}\left(Y,\tau \right)}{\partial Y^{2}}-Ma_{2}^{*}\overset{˘}{V}\left(Y,\tau \right)+\mathrm{Gr}a_{3}^{*}\overset{˘}{T}\left(Y,\tau \right). \end{array}$$

The dimensionless form of the generalized Fourier law for heat flux is:

(28) $$\begin{array}{c} {}^{C}D_{\alpha ,\beta ,a}^{\gamma }A_{4}^{*}\mathrm{Pr}\frac{\partial \overset{˘}{T}\left(Y,\tau \right)}{\partial \tau }=\frac{\partial^{2}\overset{˘}{T}\left(Y,t\right)}{\partial Y^{2}}. \end{array}$$

## 5. Results of the Problem for Artificial Replacement

This section deals with the solutions of the temperature and velocity fields with the Laplace transform method.

### 5.1. Outcome for the Temperature Field

By applying the Laplace transform to Equation (28) with conditions ${\left(12\right)}_{2}$ and ${\left(13\right)}_{3}$ and utilizing the Prabhakar fractional derivative, for the temperature field, we get

(29) $$\begin{array}{c} B_{0}{\left(1-as^{-\alpha }\right)}^{\gamma }s^{\beta }\bar{\overset{˘}{T}}\left(Y,s\right)=\frac{\partial \bar{\overset{˘}{T}}\left(Y,s\right)}{\partial Y^{2}}, \end{array}$$

which satisfies the following limitations:

(30) $$\begin{array}{c} \bar{\tilde{T}}\left(0,s\right)=0,\;\;\;\;\bar{\tilde{T}}\left(1,s\right)=\frac{1}{s}. \end{array}$$

The general solution of Equation (29) with Equation (30) is as follows:

(31) $$\begin{array}{c} \bar{\tilde{T}}\left(Y,s\right)=\frac{1}{s}\left[\frac{sinhY\sqrt{B_{0}\;s^{\beta }{\left(1-as^{-\alpha }\right)}^{\gamma }}}{sinh\sqrt{B_{0}\;s^{\beta }{\left(1-as^{-\alpha }\right)}^{\gamma }}}\right]. \end{array}$$

It is important that previous Equation can be written in the equivalent form:

(32) $$\begin{array}{c} \bar{\overset{˘}{T}}\left(Y,s\right)=\frac{1}{s}\left[\sum_{m=0}^{\infty }e^{-\left(2m+1-Y\right)\sqrt{B_{0}s^{\beta }{\left(1-as^{-\alpha }\right)}^{\gamma }}}-\sum_{m=0}^{\infty }e^{-\left(2m+1+Y\right)\sqrt{B_{0}s^{\beta }{\left(1-as^{-\alpha }\right)}^{\gamma }}}\right]. \end{array}$$

The inverse Laplace transform cannot be found simply from the Laplace transform equation. As a result, Equation (32) is represented in the series as follows:

(33) $$\begin{array}{c} \bar{\overset{˘}{T}}\left(Y,s\right)=\frac{1}{s}+\sum_{m=0}^{\infty }\sum_{l_{1}=1}^{\infty }\sum_{l_{1}=0}^{\infty }\frac{{\left(Y-2m-1\right)}^{l_{1}}{\left(B_{0}\right)}^{\frac{l_{1}}{2}}{\left(a\right)}^{l_{2}}}{l_{1}!l_{2}!\;s^{\alpha l_{2}-\frac{\beta l_{1}}{2}+1}}\frac{\Gamma (\frac{\gamma l_{1}}{2}+1)}{\Gamma (\frac{\gamma l_{1}}{2}+1-l_{2})}\\ +\sum_{m=0}^{\infty }\sum_{l_{3}=0}^{\infty }\sum_{l_{4}=0}^{\infty }\frac{{\left(-Y-2m-1\right)}^{l_{3}}{\left(B_{0}\right)}^{\frac{l_{3}}{2}}{\left(a\right)}^{l_{4}}}{l_{3}!l_{4}!\;s^{\alpha l_{4}-\frac{\beta l_{3}}{2}+1}}\frac{\Gamma (\frac{\gamma l_{3}}{2}+1)}{\Gamma (\frac{\gamma l_{3}}{2}+1-l_{4})}. \end{array}$$

Using the inverse Laplace transform of Equation (33), we have

(34) $$\begin{array}{c} \overset{˘}{T}\left(Y,\tau \right)=1+\sum_{m=0}^{\infty }\sum_{l_{1}=1}^{\infty }\sum_{l_{1}=0}^{\infty }\frac{{\left(Y-2m-1\right)}^{l_{1}}{\left(B_{0}\right)}^{\frac{l_{1}}{2}}{\left(a\right)}^{l_{2}}}{l_{1}!l_{2}!\;}\frac{t^{\alpha l_{2}-\frac{\beta l_{1}}{2}}}{\Gamma (\alpha l_{2}-\frac{\beta l_{1}}{2}+1)}\frac{\Gamma (\frac{\gamma l_{1}}{2}+1)}{\Gamma (\frac{\gamma l_{1}}{2}+1-l_{2})}\\ +\sum_{m=0}^{\infty }\sum_{l_{3}=0}^{\infty }\sum_{l_{4}=0}^{\infty }\frac{{\left(-Y-2m-1\right)}^{l_{3}}{\left(B_{0}\right)}^{\frac{l_{3}}{2}}{\left(a\right)}^{l_{4}}}{l_{3}!l_{4}!\;}\frac{t^{\alpha l_{4}-\frac{\beta l_{3}}{2}}}{\Gamma (\alpha l_{4}-\frac{\beta l_{3}}{2}+1)}\frac{\Gamma (\frac{\gamma l_{3}}{2}+1)}{\Gamma (\frac{\gamma l_{3}}{2}+1-l_{4})}. \end{array}$$

### 5.2. Outcome for the Velocity Field

By applying the Laplace transform to Equation (27) with constraints ${\left(12\right)}_{1}$ and ${13)}_{1}$, we attain

(35) $$\begin{array}{c} \left[\frac{\partial^{2}}{\partial Y^{2}}-B_{1}s^{\beta }{\left(1-as^{-\alpha }\right)}^{\gamma }-B_{2}\right]\bar{\overset{˘}{V}}\left(Y,s\right)=-B_{3}\bar{\overset{˘}{T}}\left(Y,s\right), \end{array}$$

which satisfies the following constraints:

(36) $$\begin{array}{c} \bar{\overset{˘}{V}}\left(0,s\right)=0,\;\;\;\;\bar{\overset{˘}{V}}\left(1,s\right)=0. \end{array}$$

From Equations (32), (35), and (36), we acquire the following results:

(37) $$\begin{array}{c} \bar{\overset{˘}{V}}\left(Y,s\right)=-\frac{B_{4}}{s}\left[\frac{\sum_{m=0}^{\infty }e^{-\left(2m\right)\sqrt{B_{0}s^{\beta }{\left(1-as^{-\alpha }\right)}^{\gamma }}}-\sum_{m=0}^{\infty }e^{-\left(2m+2\right)\sqrt{B_{0}s^{\beta }{\left(1-as^{-\alpha }\right)}^{\gamma }}}}{\left[1+\frac{\left(B_{1}-B_{0}\right)s^{\beta }{\left(1-as^{-\alpha }\right)}^{\gamma }}{B_{2}}\right]}\right]\\ \times \left[\sum_{n=0}^{\infty }e^{\left(Y-2n-1\right)\sqrt{B_{2}+B_{1}{\left(1-as^{-\alpha }\right)}^{\gamma }s^{\beta }}}-\sum_{n=0}^{\infty }e^{\left(-Y-2n-1\right)\sqrt{B_{2}+B_{1}{\left(1-as^{-\alpha }\right)}^{\gamma }s^{\beta }}}\right]\\ +\frac{B_{4}}{s}\left[\frac{\sum_{m=0}^{\infty }e^{-\left(2m+1-Y\right)\sqrt{B_{0}s^{\beta }{\left(1-as^{-\alpha }\right)}^{\gamma }}}-\sum_{m=0}^{\infty }e^{-\left(2m+1+Y\right)\sqrt{B_{0}s^{\beta }{\left(1-as^{-\alpha }\right)}^{\gamma }}}}{\left[1+\frac{\left(B_{1}-B_{0}\right)s^{\beta }{\left(1-as^{-\alpha }\right)}^{\gamma }}{B_{2}}\right]}\right]. \end{array}$$

The inverse Laplace transform of Equation (37) can be obtained numerically by using Tzou’s and Stehfest’s algorithms [38,39].

## 6. Results and Discussion

**Figure 2:**
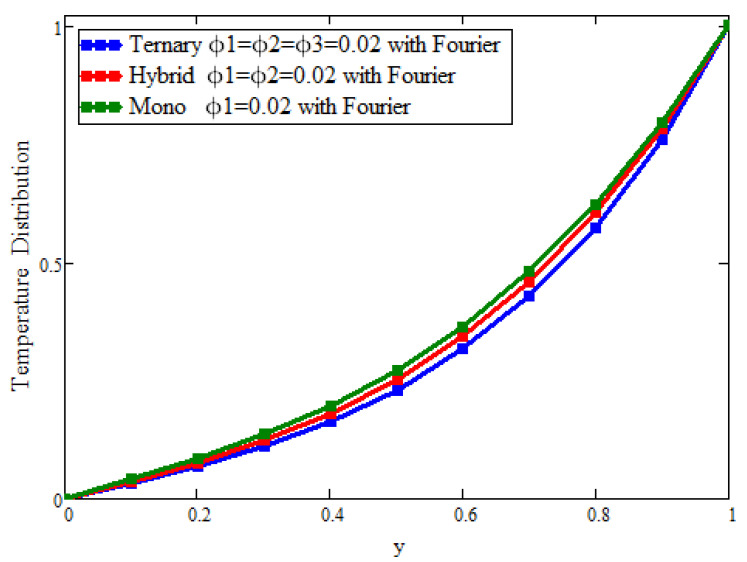
Comparison of temperature field assessments across y for the volumetric fraction of $\phi_{1}=\phi_{2}=\phi_{3}$ = 0.02, where α=β=γ = 0.5, a = 0.02, t = 1.6, and Pr = 21.

**Figure 3:**
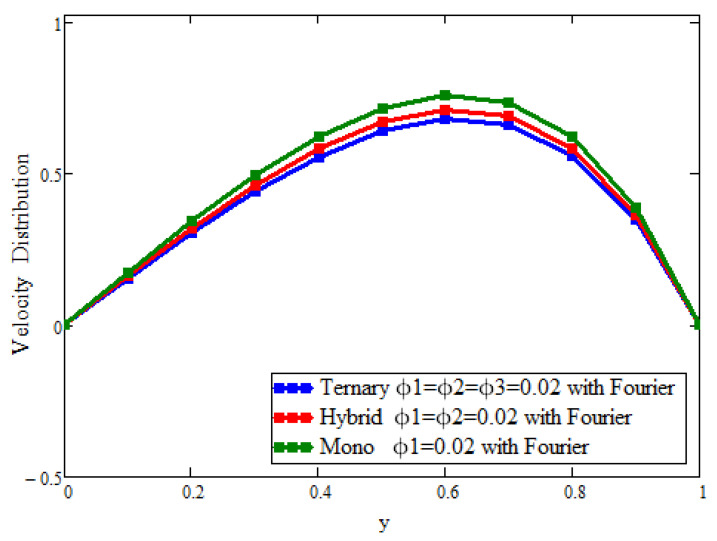
Comparison of velocity field assessments across y for the volumetric fraction of $\phi_{1}=\phi_{2}=\phi_{3}$ = 0.02, where α=β=γ = 0.5, t = 1.1, Gr = 15, Pr = 21, a = 0.02, and M = 1.2.

Figure 2 and Figure 3 present the behavior of the concentrations of the three types of nanoparticles for the temperature and velocity fields, respectively. It is clear in Figure 2 that the temperature can be enhanced to its maximum, unlike with hybrid and mono-nanoparticles. Due to the increase in the concentration of ternary nanoparticles, the thermal conductivity and, hence, the temperature increase. Figure 3 depicts the outcomes for the velocity to see the impact of the volume fraction ($\phi_{1}$ = $\phi_{2}$ = $\phi_{3}$ = 0.02). It is found that the velocity is a decreasing function of the concentration of the nanoparticles, and the maximum decline can be observed, unlike with hybrid and mono-nanoparticles. Physically, this is due to fact that increasing the concentration causes the fluid to become more thick; the space between the layers is reduced and, ultimately, the fluid flows slowly.

**Figure 4:**
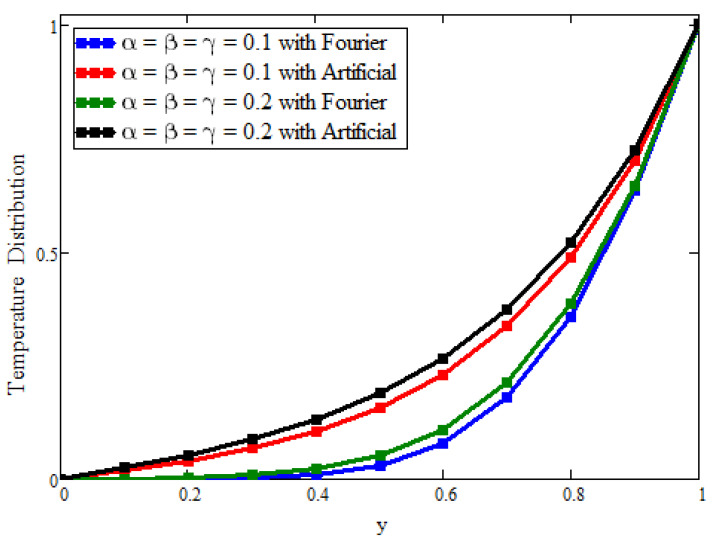
The effects of fractional parameters on the temperature field. Here, a = 0.02, t = 0.4, Pr = 21, $\phi_{1}=0.01,\phi_{2}=0.02,$ and $\phi_{3}$ = 0.03.

**Figure 5:**
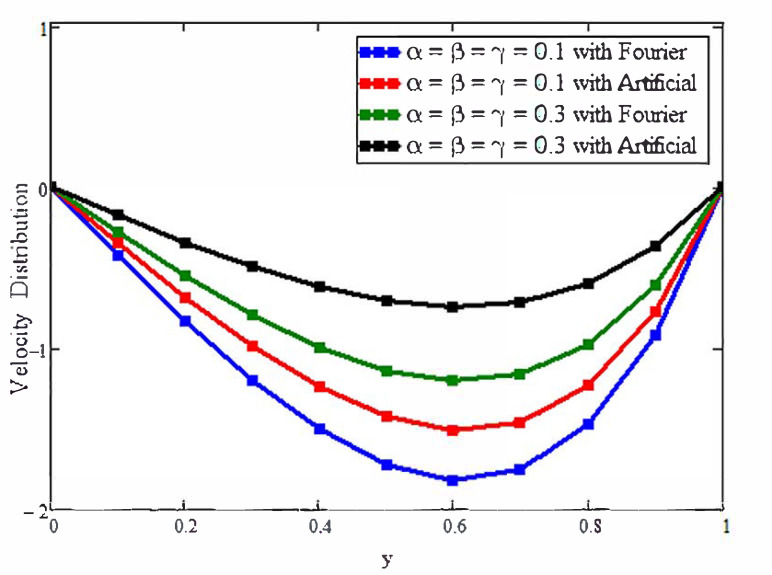
The effects of fractional parameters on the velocity field. Here, a = 0.02, t = 0.2, Pr = 21, Gr = 15, M = 1.2, $\phi_{1}=0.01,\phi_{2}=0.02,$ and $\phi_{3}$ = 0.03.

Figure 4 presents the issue of modeling with the fractional derivative. This figure is plotted for the solutions that were obtained artificially and with the Fourier law. By fixing the flow parameters as constant and varying the values of the fractional parameters, it is evident that the solutions based on the Fourier law are efficient and exhibit more memory in comparison with the solutions obtained through replacement. A similar behavior is observed for velocity, as shown in Figure 5. Physically, the fractional operators are responsible for the memory of the fluid’s properties for different values of the fractional parameters at different times.

**Figure 6:**
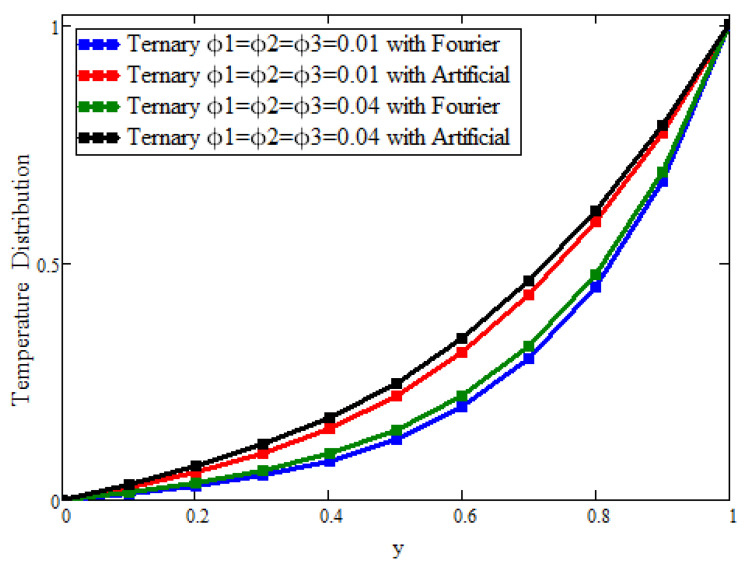
The effect of the volumetric fraction on the temperature field. Here, Pr = 21, γ=0.7,α=0.7,β=0.7, a = 0.02, and t = 0.3.

**Figure 7:**
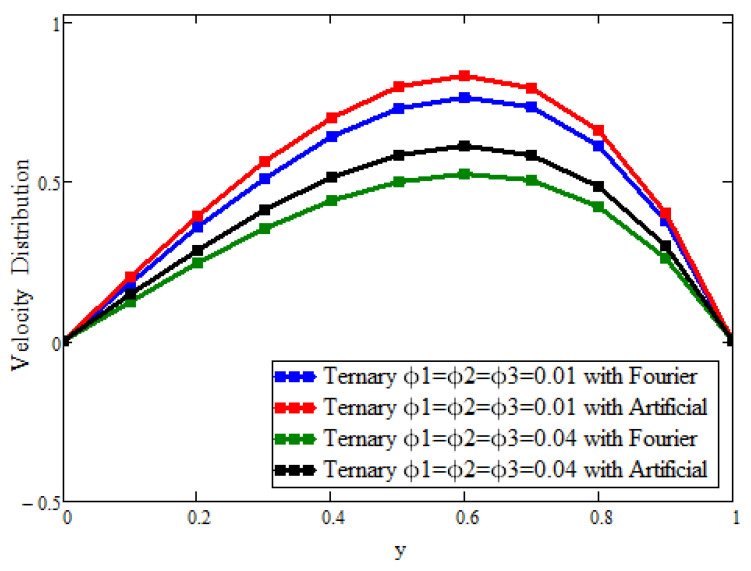
The effect of the volumetric fraction on the velocity field. Here, Pr = 21, a = 0.02, t = 2, α=0.7,β=0.7,γ=0.7, Gr = 15, and M = 1.2.

Figure 6 shows only the effects of ternary nanoparticles with the Fourier law and replacement. Taking $\phi_{1}$ = $\phi_{2}$ = $\phi_{3}$ = 0.01 for the solutions, it was found that the ternary nanoparticles with the Fourier law predicted a greater enhancement in the temperature than that predicted in the artificial case. On the other hand, by increasing the concentration from 0.01 to 0.04, the temperature also increased, which supported the physical reasoning of the fluid temperature. Figure 7 plots only the effects of the ternary nanoparticles with the Fourier law and replacement. Taking $\phi_{1}$ = $\phi_{2}$ = $\phi_{3}$ = 0.01 for the solutions, it was found that the ternary nanoparticles with the Fourier law predicted a greater decline in the velocity than that predicted in the artificial case. On the other hand, by increasing the concentration from 0.01 to 0.04, the velocity was also decreased, which supported the physical reasoning of the fluid velocity.

## 7. Conclusions

This study focuses on the numerical solutions of fractional partial differential equations that appear in phenomena. An approach that uses Prabhakar fractional models with a Laplace transform is used to investigate unsteady MHD convective streams of incompressible viscous fluids in a moving frame with a non-Newtonian fluid in a turbulent channel with ternary nanoparticles and Prabhakar fractional derivatives on the boundary. Therefore, the thermal transport model is based on the generalized fractional Fourier law of the thermal flux. By using the Mathcad software, some of the physical implications of the flow characteristics were examined. Graphical representations of two components, velocity and temperature, were created. It was discovered that the thermal transport’s damping has a significant impact on the fluid’s temperature and velocity.

- The heat transfer properties of ternary nanoparticles are superior to those of fluids, mono-nanoparticles, and hybrid nanoparticles.
- It is evident that fractional modeling based on the Fourier law is efficient and suitable in comparison with replacement.
- The Prabhakar fractional approach is responsible for the better memory of the function due to the generalized Mittag–Leffer kernel of the three parameters.

## Figures and Tables

**Table 1:** Thermo-physical properties of ternary nanoparticles [34].

Ternary Nanoparticles
$\mu_{mnf}=\mu_{f}{\left(1-\left(\phi_{Cu}+\phi_{Ag}+\phi_{TiO_{2}}\right)\right)}^{-2.5}$
$\rho_{mnf}=\left(1-\left(\phi_{mnf}\right)\rho_{f}\right)+\phi_{Cu}\rho_{Cu}+\phi_{Ag}\rho_{Ag}+\phi_{TiO_{2}}\rho_{TiO_{2}}$
${\left(\rho C_{p}\right)}_{mnf}=(1-\left(\phi_{mnf}\right){\left(\rho C_{p}\right)}_{f}+\phi_{Cu}{\left(\rho C_{p}\right)}_{Cu}+\phi_{Ag}{\left(\rho C_{p}\right)}_{Ag}+\phi_{TiO_{2}}{\left(\rho C_{p}\right)}_{TiO_{2}}$
${\left(\rho \beta_{T}\right)}_{mnf}=\left(1-\phi_{mnf}\right){\left(\rho \beta_{T}\right)}_{f}+\phi_{Cu}{\left(\rho \beta_{T}\right)}_{Cu}+\phi_{Ag}{\left(\rho \beta_{T}\right)}_{Ag}+\phi_{TiO_{2}}{\left(\rho \beta_{T}\right)}_{TiO_{2}}$
$\frac{\sigma_{mnf}}{\sigma_{f}}=1+\frac{3(\frac{\phi_{Cu}\sigma_{Cu}+\phi_{Ag}\sigma_{Ag}+\phi_{TiO_{2}}\sigma_{TiO_{2}}}{\sigma_{f}}-\phi_{mnf})}{\left(\frac{\phi_{Cu}\sigma_{Cu}+\phi_{Ag}\sigma_{Ag}+\phi_{TiO_{2}}\sigma_{TiO_{2}}}{\phi_{mnf}\sigma_{f}}+2\right)-\left(\frac{\phi_{Cu}\sigma_{Cu}+\phi_{Ag}\sigma_{Ag}+\phi_{TiO_{2}}\sigma_{TiO_{2}}}{\sigma_{f}}-\phi_{mnf}\right)}$
$\frac{k_{mnf}}{k_{f}}=\frac{\frac{\phi_{Cu}k_{Cu}+\phi_{Ag}k_{Ag}+\phi_{TiO_{2}}k_{TiO_{2}}}{\phi_{mnf}}+2k_{f}+2\left(\phi_{Cu}k_{Cu}+\phi_{Ag}k_{Ag}+\phi_{TiO_{2}}k_{TiO_{2}}\right)-2k_{f}\phi_{mnf}}{\frac{\phi_{Cu}k_{Cu}+\phi_{Ag}k_{Ag}+\phi_{TiO_{2}}k_{TiO_{2}}}{\phi_{mnf}}+2k_{f}+\left(\phi_{Cu}k_{Cu}+\phi_{Ag}k_{Ag}+\phi_{TiO_{2}}k_{TiO_{2}}\right)-k_{f}\phi_{mnf}}$

**Table 2:** Thermo-physical properties of nanoparticles and the base fluid from [35,36].

Material	Base Fluid: Kerosene Oil	Silver (Ag)	Copper (Cu)	Titanium Dioxide (TiO_2_)
ρ (kg/m^3^)	783	10500	8993	4250
$C_{p}$ (J/kg·K)	2090	235	385	686.20
*k* (W/m·K)	0.145	429	401	8.9538
σ (s/m)	$21\times 10^{-6}$	$3.6\times 10^{7}$	$59.6\times 10^{6}$	$2.6\times 10^{6}$
$\beta \times 10^{-5}$ (1/K)	99	1.89	1.67	0.90
Pr	21			

## Data Availability

All data are available within this manuscript.

## Abbreviations

The following abbreviations are used in this manuscript:

Pr	Dimensionless Prandtl number
Gr	Dimensionless Groshof number
M	Magnetic field parameter
$\overset{˘}{k}$	Thermal conductivity (m/WK)
$\overset{˘}{\theta }$	Dimensionless temperature
ρ	Fluid density (kg/m^3^)
σ	Electrical conductivity
ν	Kinematic viscosity (m^2^/s)
*t*	Time (s)
μ	Dynamic viscosity (kg/ms)
$C_{p}$	Specific heat (J/kg K)
$\left(\rho C_{p}\right)$	Heat capacitance (J/kg m^3^)
$\overset{˘}{T}$	Temperature (K)
${\overset{˘}{T}}_{\infty }$	Ambient temperature (K)
${\overset{˘}{T}}_{w}$	Surface temperature (K)
$\overset{˘}{u},\overset{˘}{v}$	Velocity component (m/s)
α,β,γ	Fractional parameters

## References

[1] Bräuer F., Trautner E., Hasslberger J., Cifani P., Klein M. (2021). Turbulent bubble-laden Channel flow of power-law fluids: A direct numerical simulation study. Fluids.

[2] Dippolito A., Calomino F., Alfonsi G., Lauria A. (2021). Flow resistance in open channel due to vegetation at reach scale: A review. Water.

[3] Zheng Y., Yang H., Mazaheri H., Aghaei A., Mokhtari N., Afrand M. (2021). An investigation on the influence of the shape of the vortex generator on fluid flow and turbulent heat transfer of hybrid nanofluid in a channel. J. Therm. Anal. Calorim..

[4] Asjad M.I., Ikram M.D., Sarwar N., Muhammad T., Sivasankaran S., Subaihi S.A.A. (2022). Analysis of fractional bioconvection with hybrid nanoparticles in Channel flow. Math. Probl. Eng..

[5] Nadeem S., Ahmad S., Khan M.N. (2021). Mixed convection flow of hybrid nanoparticle along a Riga surface with Thomson and Troian slips condition. J. Therm. Anal. Calorim..

[6] Choi S.U.S., Eastman J.A. (1995). Enhancing Thermal Conductivity of Fluids with Nanoparticles.

[7] Eastman J.A., Choi S.U.S., Li S., Yu W., Thompson L.J. (2021). Anomalously increased effective thermal conductivities of ethylene glycol-based nanofluids containing copper nanoparticles. Appl. Phys. Lett..

[8] Asjad M.I., Basit A., Iqbal A., Shah N.A. (2021). Advances in transport phenomena with nanoparticles and generalized thermal process for vertical plate. Phys. Scr..

[9] Reddy M.G., Shehzad S.A. (2021). Molybdenum disulfide and magnesium oxide nanoparticle performance on micropolar Cattaneo-Christov heat flux model. Appl. Math. Mech..

[10] Izadi M. (2020). Effects of porous material on transient natural convection heat transfer of nano-fluids inside a triangular chamber. Chin. J. Chem. Eng..

[11] Saeed N., Sarwar N., Riaz A. (2021). The MHD effect on a fractional Casson nanofluid with constant boundary conditions and the generalized Mittag-Leffler Kernel. Comput. J. Comb. Math..

[12] Babazadeh H., Shah Z., Ullah I., Kumam P., Shafee A. (2021). Analysis of hybrid nanofluid behavior within a porous cavity including Lorentz forces and radiation impacts. J. Therm. Anal. Calorim..

[13] Nadeem S., Abbas N., Malik M.Y. (2020). Inspection of hybrid based nanofluid flow over a curved surface. Comput. Methods Programs Biomed..

[14] Waini I., Ishak A., Grosan T., Pop I. (2020). Mixed convection of a hybrid nanofluid flow along a vertical surface embedded in a porous medium. Int. Commun. Heat Mass Transf..

[15] Asadi A., Alarifi I.M., Foong L.K. (2020). An experimental study on characterization, stability and dynamic viscosity of CuO-TiO_2_/water hybrid nanofluid. J. Mol. Liq..

[16] Huminic G., Huminic A. (2020). Entropy generation of nanofluid and hybrid nanofluid flow in thermal systems: A review. J. Mol. Liq..

[17] Asjad M.I., Sarwar N., Hafeez M.B., Sumelka W., Muhammad T. (2021). Advancement of non-newtonian fluid with hybrid nanoparticles in a convective channel and prabhakar’s fractional derivative—Analytical solution. Fractal Fract..

[18] Shah N.A., Wakif A., El-Zahar E.R., Thumma T., Yook S.J. (2022). Heat transfers thermodynamic activity of a second-grade ternary nanofluid flow over a vertical plate with Atangana-Baleanu time-fractional integral. Alex. Eng. J..

[19] Guedri K., Khan A., Gul T., Mukhtar S., Alghamdi W., Yassen M.F., Tag Eldin E. (2022). Thermally dissipative flow and Entropy analysis for electromagnetic trihybrid nanofluid flow past a stretching surface. ACS Omega.

[20] Sahoo R.R., Kumar V. (2020). Development of a new correlation to determine the viscosity of ternary hybrid nanofluid. Int. Commun. Heat Mass Transf..

[21] Mousavi S.M., Esmaeilzadeh F., Wang X.P. (2019). Effects of temperature and particles volume concentration on the thermophysical properties and the rheological behavior of CuO/MgO/TiO_2_ aqueous ternary hybrid nanofluid. J. Therm. Anal. Calorim..

[22] Raju C.S.K., Ahammad N.A., Sajjan K., Shah N.A., Yook S.J., Kumar M.D. (2022). Nonlinear movements of axisymmetric ternary hybrid nanofluids in a thermally radiated expanding or contracting permeable Darcy Walls with different shapes and densities: Simple linear regression. Int. Commun. Heat Mass Transf..

[23] Ramadhan A.I., Azmi W.H., Mamat R., Hamid K.A., Norsakinah S. (2019). Investigation on stability of tri-hybrid nanofluids in water-ethylene glycol mixture. In IOP Conf. Ser. Mater. Sci. Eng..

[24] West B.J., Bologna M., Grigolini P. (2003). Fractional Fourier transforms. Phys. Fractal Oper..

[25] Kumar S., Kumar D., Abbasbandy S., Rashidi M.M. (2014). Analytical solution of fractional Navier-Stokes equation by using modified Laplace decomposition method. Ain Shams Eng. J..

[26] Diethelm K., Ford N.J. (2002). Analysis of fractional differential equations. J. Math. Anal. Appl..

[27] Sene N. (2020). Second-grade fluid model with Caputo-Liouville generalized fractional derivative. Chaos Solitons Fractals.

[28] Mozafarifard M., Toghraie D. (2020). Time-fractional subdiffusion model for thin metal films under femtosecond laser pulses based on Caputo fractional derivative to examine anomalous diffusion process. Int. J. Heat Mass Transf..

[29] Reyaz R., Mohamad A.Q., Lim Y.J., Saqib M., Shafie S. (2022). Analytical solution for impact of Caputo-Fabrizio fractional derivative on MHD casson fluid with thermal radiation and chemical reaction effects. Fractal Fract..

[30] Haq S.U., Khan M.A., Khan Z.A., Ali F. (2020). MHD effects on the channel flow of a fractional viscous fluid through a porous medium: An application of the Caputo-Fabrizio time-fractional derivative. Chin. J. Phys..

[31] Thabet S.T., Abdo M.S., Shah K., Abdeljawad T. (2020). Study of transmission dynamics of COVID-19 mathematical model under ABC fractional order derivative. Results Phys..

[32] Sarwar N., Asjad M.I., Sitthiwirattham T., Patanarapeelert N., Muhammad T. (2021). A Prabhakar fractional approach for the convection flow of casson fluid across an oscillating surface based on the generalized Fourier law. Symmetry.

[33] Shah N.A., Fetecau C., Vieru D. (2021). Natural convection flows of Prabhakar-like fractional Maxwell fluids with generalized thermal transport. J. Therm. Anal. Calorim..

[34] Shafie S., Saqib M., Khan I., Qushairi A. (2019). Mixed convection flow of brinkman type hybrid nanofluid based on Atangana-Baleanu fractional model. J. Phys. Conf. Ser..

[35] Asjad M.I., Ikram M.D., Ali R., Baleanu D., Alshomrani A.S. (2020). New analytical solutions of heat transfer flow of clay-water base nanoparticles with the application of novel hybrid fractional derivative. Therm. Sci..

[36] Kumar M.A., Reddy Y.D., Rao V.S., Goud B.S. (2021). Thermal radiation impact on MHD heat transfer natural convective nano fluid flow over an impulsively started vertical plate. Case Stud. Therm. Eng..

[37] Tanveer M., Ullah S., Shah N.A. (2021). Thermal analysis of free convection flows of viscous carbon nanotubes nanofluids with generalized thermal transport: A Prabhakar fractional model. J. Therm. Anal. Calorim..

[38] Stehfest H. (1970). Algorithm 368: Numerical inversion of Laplace transforms [D5]. Commun. ACM.

[39] Tzou D.Y. (2014). Macro-to Microscale Heat Transfer: The Lagging Behavior.

